# Data Poisoning Vulnerabilities Across Health Care Artificial Intelligence Architectures: Analytical Security Framework and Defense Strategies

**DOI:** 10.2196/87969

**Published:** 2026-01-23

**Authors:** Farhad Abtahi, Fernando Seoane, Ivan Pau, Mario Vega-Barbas

**Affiliations:** 1 Department of Clinical Science, Intervention and Technology Karolinska Institutet Huddinge, Stockholm Sweden; 2 Department of Biomedical Engineering and Health Systems KTH Royal Institute of Technology Huddinge, Stockholm Sweden; 3 Department of Clinical Physiology Karolinska University Hospital Huddinge, Stockholm Sweden; 4 Department of Medical Technology Karolinska University Hospital Stockholm Sweden; 5 Department of Textile Technology University of Borås Borås, Västra Götaland Sweden; 6 ETSIS de Telecomunicación Universidad Politécnica de Madrid Madrid, Madrid Spain

**Keywords:** artificial intelligence, health care security, data poisoning, backdoor attacks, clinical decision support, federated learning, large language models, medical imaging, patient safety, AI governance

## Abstract

**Background:**

Health care artificial intelligence (AI) systems are increasingly integrated into clinical workflows, yet remain vulnerable to data-poisoning attacks. A small number of manipulated training samples can compromise AI models used for diagnosis, documentation, and resource allocation. Existing privacy regulations, including the Health Insurance Portability and Accountability Act and the General Data Protection Regulation, may inadvertently complicate anomaly detection and cross-institutional auditing, thereby limiting visibility into adversarial activity.

**Objective:**

This study provides a comprehensive threat analysis of data poisoning vulnerabilities across major health care AI architectures. The goals are to (1) identify attack surfaces in clinical AI systems, (2) evaluate the feasibility and detectability of poisoning attacks analytically modeled in prior security research, and (3) propose a multilayered defense framework appropriate for health care settings.

**Methods:**

We synthesized empirical findings from 41 key security studies published between 2019 and 2025 and integrated them into an analytical threat-modeling framework specific to health care. We constructed 8 hypothetical yet technically grounded attack scenarios across 4 categories: (1) architecture-specific attacks on convolutional neural networks, large language models, and reinforcement learning agents (scenario A); (2) infrastructure exploitation in federated learning and clinical documentation pipelines (scenario B); (3) poisoning of critical resource allocation systems (scenario C); and (4) supply chain attacks affecting commercial foundation models (scenario D). Scenarios were aligned with realistic insider-access threat models and current clinical deployment practices.

**Results:**

Multiple empirical studies demonstrate that attackers with access to as few as 100-500 poisoned samples can compromise health care AI systems, with attack success rates typically ≥60%. Critically, attack success depends on the absolute number of poisoned samples rather than their proportion of the training corpus, a finding that fundamentally challenges assumptions that larger datasets provide inherent protection. We estimate that detection delays commonly range from 6 to 12 months and may extend to years in distributed or privacy-constrained environments. Analytical scenarios highlight that (1) routine insider access creates numerous injection points across health care data infrastructure, (2) federated learning amplifies risks by obscuring attribution, and (3) supply chain compromises can simultaneously affect dozens to hundreds of institutions. Privacy regulations further complicate cross-patient correlation and model audit processes, substantially delaying the detection of subtle poisoning campaigns.

**Conclusions:**

Health care AI systems face significant security challenges that current regulatory frameworks and validation practices do not adequately address. We propose a multilayered defense strategy that combines ensemble disagreement monitoring, adversarial testing, privacy-preserving yet auditable mechanisms, and strengthened governance requirements. Ensuring patient safety may require a shift from opaque, high-performance models toward more interpretable and constraint-driven architectures with verifiable robustness guarantees.

## Introduction

Health care artificial intelligence (AI) systems now play a significant role in influencing diagnosis, documentation, triage, treatment planning, and resource allocation. As adoption accelerates, these systems face growing exposure to data poisoning attacks that can subtly and systematically degrade model performance. Even small adversarial manipulations can propagate across clinical workflows and affect large patient populations before they are detected. Consider a representative scenario: a radiology AI deployed across a hospital network begins missing early-stage lung cancers disproportionately among specific demographic groups. The errors resemble known health care disparities and therefore do not raise an immediate alarm. Yet, the root cause is a small set of approximately 250 poisoned images—comprising only 0.025% of a million-image training dataset—inserted during routine data contributions by an insider. Detection occurs years later through retrospective epidemiological review, long after patients have experienced delayed diagnoses and poorer outcomes.

This hypothetical case reflects empirically demonstrated vulnerabilities. Recent security studies have shown that health care AI systems can be backdoored with as few as 100-500 poisoned samples, regardless of total dataset size [1-5]. Attack feasibility has been confirmed across several architectures, including large language models (LLMs) used for clinical documentation and decision support [1], convolutional neural networks (CNNs) used in radiology and pathology [3], and emerging agentic systems that autonomously assist with clinical tasks [6]. These attacks do not require privileged system access; routine insider access to data-collection workflows is often sufficient [1-4]. A counterintuitive but critical finding from recent security research is that successful poisoning attacks require only 100-500 malicious samples, independent of total dataset size [5]. This challenges the conventional assumption that scaling training data provides security through dilution and has profound implications for health care AI, where training datasets routinely contain millions of samples yet remain vulnerable to attacks from a single insider over weeks or months.

Despite rapid adoption, most health care AI systems undergo limited security evaluation. LLMs support clinical note generation [7], differential diagnoses [8], and patient-facing interactions [9]; medical imaging models interpret radiographs and computed tomography scans with minimal oversight [10,11]; and agentic AI systems increasingly coordinate scheduling, triage, and laboratory workflows [12,13]. Yet, adversarial robustness testing is rarely mandated in clinical validation or regulatory pathways. Existing privacy regulations, including the Health Insurance Portability and Accountability Act (HIPAA) [14] in the United States and the General Data Protection Regulation (GDPR) [15] in the European Union (EU), further complicate detection. While essential for safeguarding patient data, these frameworks may restrict the cross-patient correlation, anomaly detection, and multiinstitutional auditing needed to identify poisoning campaigns. Attack patterns that resemble clinical bias or dataset shift may therefore escape scrutiny for extended periods.

Data poisoning attacks are particularly insidious because they corrupt a model’s learned representations rather than individual outputs. Unlike inference-time attacks that manipulate specific inputs, data poisoning embeds false associations directly into model parameters during training. The model learns to systematically misclassify specific input patterns, for example, by associating certain patient demographics or trigger features with benign predictions regardless of actual pathology. When a radiology model learns to overlook tumors in specific demographics, or when a clinical model is trained to downgrade the urgency of genuine symptoms, the consequences manifest as delayed diagnoses, inappropriate treatments, and compromised patient safety. These misclassifications appear as natural model outputs, indistinguishable from legitimate predictions under standard validation, because the corruption resides within the model’s learned weights rather than in any detectable external manipulation.

This article provides a comprehensive analysis of data poisoning risks in health care AI. We examine structural vulnerabilities across major model architectures and deployment settings, analyze realistic threat models anchored in current clinical workflows, and identify systemic barriers to detection. Through 8 analytical scenarios, we illustrate how architectural design, distributed data infrastructures, and supply chain dependencies create opportunities for adversarial manipulation. Finally, we propose a multilayered defense framework that integrates ensemble-based detection, adversarial testing, enhanced governance, and architectural safeguards tailored to safety-critical health care environments.

Our novel contributions are (1) to our best knowledge, the first systematic threat analysis adapting data poisoning research from general machine learning (ML) security to health care–specific contexts, accounting for clinical workflows, regulatory constraints, and patient safety requirements; (2) 8 analytically constructed attack scenarios (A1-D1) demonstrating how empirically validated attacks apply to realistic health care deployment settings across all major AI architectures; (3) identification of the privacy regulation paradox, wherein HIPAA and GDPR protections essential for patient privacy simultaneously create detection blind spots that attackers can exploit; (4) scenario-specific application of the MEDLEY (Medical Ensemble Diagnostic system with Leveraged Diversity) ensemble disagreement framework to health care AI, with concrete detection protocols tailored to clinical settings (Table 1); and (5) analysis of supply chain vulnerabilities in health care AI, identifying how single-vendor compromises can create systemic risks across dozens to hundreds of institutions. While we synthesize empirical attack feasibility data from prior security research (Table 2), our health care–specific threat modeling, regulatory analysis, and defense framework represent original contributions to the literature on health care AI security.

**Table 1:** MEDLEY^a^ framework application to attack scenarios^b^.

Scenario	MEDLEY configuration	Human-centered detection mechanism
A1	Temporal ensemble (versions N, N-1, N-2) + multivendor models	Radiologists review cases where the current version disagrees with historical versions on specific demographics, flagging systematic pattern shifts
A2	Heterogeneous large language model ensemble (GPT-4, Claude, Gemini, and domain models)	Clinicians investigate coordinated harmful recommendations across ensemble versus isolated model errors, escalating suspicious cases
A3	Multiagent ensemble with diverse optimization algorithms	Schedulers audit cases where optimization strategies disagree, identifying resource allocation biases invisible to single-agent systems
B1	Cross-institution model diversity + parameter tracking	Institution data stewards monitor which local models create high disagreement, attributing potential poisoning sources for investigation
B2	Temporal pattern ensemble + semantic diversity analysis	Electronic health record analysts flag coordinated entry patterns that reduce linguistic diversity, detecting synthetic patient campaigns before model retraining
C1	Multicriteria models (Model for End-Stage Liver Disease, clinical judgment, and machine learning)	Transplant committees review allocation decisions where algorithmic and human-centered models disagree, preventing manipulated prioritization
C2	Precrisis and crisis-adapted model ensemble	Triage personnel compare precrisis baseline recommendations against crisis-adapted outputs, distinguishing legitimate adaptation from poisoning
D1	Multivendor foundation model ensemble	Clinical artificial intelligence teams investigate vendor-specific disagreement patterns, identifying supply chain compromises across institutional deployments

^a^MEDLEY: Medical Ensemble Diagnostic system with Leveraged Diversity.

^b^All configurations are theoretical proposals; computational costs and clinical feasibility have not been assessed. Validation status: unvalidated.

**Table 2:** Analytical attack scenarios for health care artificial intelligence systems^a,b^.

Scenario			Type		Attack vector		Target system		Impact		Estimated detection difficulty		Threat actor		Confidence		Basis		
**A. Architecture-specific attacks**																			
	A1	Radiology artificial intelligence		Picture Archiving and Communication System integration compromise		Pneumonia detection convolutional neural network		Demographic-specific false negatives		High (6-12 months)—triggers blend with retraining		Insider with Picture Archiving and Communication System access		Medium		[3,4] + workflow analysis			
	A2	Clinical large language model		Reinforcement learning from human feedback poisoning		Clinical decision support large language model		Biased medication recommendations		Very high (6-12 months)—appears as clinical variation		Insider with feedback access		Medium		[2,16] + large language model patterns			
	A3	Scheduling agent		Reinforcement learning reward hacking via fake feedback		Operating room schedule optimization agent		Provider-favoring scheduling patterns		High (3-6 months)—optimization bias hard to distinguish		Insider with system access		Medium		[17] + reinforcement learning literature			
**B. Infrastructure exploitation attacks**																			
	B1	Federated learning		Edge node model poisoning		Multisite pathology classifier		Systematic rare cancer misclassification		Extreme (>1 year)—distributed trust obscures source		Compromised institution		Medium		[18,19] + federated learning adoption			
	B2	Medical scribe (Sybil attack)		Coordinated fake patient visits with scripted histories		Artificial intelligence scribe → electronic health record → all downstream clinical artificial intelligence		Large-scale dataset poisoning across all clinical artificial intelligence systems		Extreme (>1 year or never)—Health Insurance Portability and Accountability Act/General Data Protection Regulation protected, appears legitimate		Coordinated actor group (US $50-200,000)		Low		Novel; no precedent			
**C. Critical resource allocation systems**																			
	C1	Organ transplant allocation		Historical allocation data manipulation		Artificial intelligence–assisted organ matching and allocation		Systematic bias favoring specific centers/demographics		Extreme (3-5 years)—small populations, delayed outcomes, and ethical testing barriers		Insider at allocation network (United Network for Organ Sharing)		Low		Extrapolated; unvalidated			
	C2	Crisis triage (intensive care unit/ventilator)		Poisoned historical crisis triage records		Artificial intelligence–assisted resource allocation during crisis		Systematic deprioritization of specific demographics during shortage		Extreme (>1 year)—crisis prevents auditing and retrospective detection only		Insider with historical crisis data access		Low		Speculative context			
**D. Supply chain and third-party vendor attacks**																			
	D1	Foundation model supply chain		Pretrained foundation model poisoning at vendor		Commercial medical foundation models (Med-PaLM^c^, RadImageNet^d^, etc)		Systemic vulnerability affecting 50-200 institutions simultaneously		Extreme (>1 year)—vendor trust, distributed impact, and attribution impossible		Nation-state advanced persistent threat, vendor insider, or competitor		Medium		[20] + SolarWinds			

^a^Scenarios organized by attack surface category. Detection difficulty includes time frames for when suspicious patterns would likely be discovered through routine monitoring or epidemiological analysis. “Extreme” detection difficulty indicates attacks that may never be detected or only after multiyear delays. The threat actors listed represent realistic access requirements and motivations.

^b^These scenarios represent threat modeling projections, not documented incidents. Confidence levels: high, directly supported by health care–relevant empirical studies; medium, supported by analogous studies in related domains; low, extrapolated with significant uncertainty.

^c^Med-PaLM: Medical Pathways Language Model.

^d^RadImageNet: Radiology ImageNet.

## Methods

### Analytical Framework

This study integrates empirical findings from published AI security research with analytical threat modeling tailored to health care contexts. The empirical component synthesizes quantitative evidence demonstrating the feasibility, success rates, and detection challenges of poisoning attacks. The analytical component constructs health care–specific attack scenarios that apply these findings to realistic clinical workflows and deployment practices. Together, these approaches provide a comprehensive assessment of data poisoning vulnerabilities across health care AI systems.

### Literature Identification and Evidence Synthesis

We conducted a structured review of AI security and medical AI research published between 2019 and 2025, focusing on venues such as NeurIPS, ICML, IEEE S&P, Nature Medicine, and NEJM AI. Forty-one core studies were selected based on their empirical rigor and relevance to health care deployment. Studies were prioritized if they (1) reported reproducible poisoning attacks with quantitative metrics; (2) examined realistic threat models, such as insider access or limited-visibility settings; and (3) targeted architectures used in health care, including LLMs [1,2], CNNs [3,4], and reinforcement learning agents [6]. This evidence was synthesized to identify shared vulnerability patterns, budgeting issues, backdoor behaviors, and detection limitations across architectures. Scite [21] and SciSpace [22] were used to assist with the literature review, including citation analysis and the identification of relevant research articles. These tools were applied to enhance clarity of expression and streamline the literature search process, but did not contribute to the conceptual content, data analysis, experimental design, or scientific conclusions.

### Architecture Classification

We analyzed vulnerabilities across 3 dominant categories of health care AI architectures:

- Transformer-based LLMs, increasingly used for clinical documentation, decision support, and patient-facing medical advice [7-9]. Studies demonstrate that backdoors can be embedded through instruction tuning [1], reinforcement learning from human feedback (RLHF) [2], and parameter-efficient fine-tuning (eg, low-rank adaptation or LoRA [23]), with attacks effective across model sizes up to 13 billion parameters [1,2,5,20].
- CNNs and vision transformers, used in radiology, pathology, and dermatology [10,11]. Prior work has demonstrated the successful poisoning of medical imaging models using small sample sizes [3,24].
- Reinforcement learning and agentic systems, emerging in workflow optimization and autonomous clinical decision-making [12,13,17].

Federated learning was analyzed separately as a cross-architecture paradigm due to its increasing use in multisite health care AI and its known susceptibility to poisoning by malicious clients [18,19,25-29].

### Threat Model Construction

Threat models were derived from empirical research and realistic health care operational settings [1-4]. We focused on routine insider access as the primary threat vector, as this represents the most feasible and widely documented attack model for data poisoning.

*Attacker capabilities* include the following:

- Ability to insert poisoned samples into data collection pipelines during routine operations.
- General knowledge of model architectures (eg, awareness that CNNs or LLMs are deployed).
- Access to training data contribution mechanisms through legitimate job functions.

*Attacker constraints* include the following:

- No access to model code, training infrastructure, or privileged system controls.
- No capacity to modify deployment systems or inference pipelines.
- Limited to data manipulation within their authorized access scope.

*Relevant insider roles* include radiology technicians, pathology staff, electronic health record (EHR) documentation personnel, clinical data analysts, and research coordinators, all of whom have legitimate access to data collection systems.

*Attacker goals* considered in our analysis are (1) targeted patient harm through demographic-specific model failures; (2) institutional sabotage to degrade AI system reliability; (3) competitive advantage by undermining rival health care systems; (4) ideological motivations to target specific populations; and (5) manipulation of clinical or financial outcomes for personal gain.

*Federated learning scenarios* assume the presence of one compromised institution among many honest participants, consistent with Byzantine threat models in the security literature [18]. Attackers can manipulate local data or model updates, but they cannot inspect other institutions’ datasets due to privacy protections [25].

### Regulatory Framework Assessment

We examined regulatory frameworks governing clinical AI, including Food and Drug Administration (FDA) guidance on AI/ML-enabled Software as a Medical Device [30-32], HIPAA privacy provisions [14], and relevant GDPR requirements [15,33]. The assessment focused on identifying the following:

- Gaps in mandated adversarial testing.
- Limitations in auditing and anomaly detection.
- Privacy-driven constraints on cross-institutional monitoring.
- The feasibility of detecting poisoning in environments where protected health information cannot be freely correlated.

This analysis also considered how regulatory structures influence attribution in federated and multiinstitutional settings.

### Defense Mechanism Evaluation

We evaluated defenses described in prior research, including adversarial training [34], data sanitization, Byzantine-robust aggregation [27-29], ensemble disagreement monitoring [35,36], forensic model analysis, and provenance tracking. Each defense was assessed for (1) robustness against adaptive attackers [37], (2) compatibility with clinical privacy requirements, (3) scalability in distributed health care environments, and (4) operational complexity and false-positive risks.

Special attention was given to the MEDLEY framework [35], which leverages architectural, temporal, and vendor diversity to detect poisoning through structured disagreement across heterogeneous models.

### Impact Assessment Methodology

Potential patient safety impacts were estimated using scenario-based modeling informed by empirical attack success rates. We examined the following:

- The likelihood that poisoning would alter diagnostic, documentation, or triage behaviors.
- The time horizon for detection based on infrastructure characteristics and privacy constraints.
- Downstream effects on clinical outcomes using conservative assumptions about partial compromise, demographic targeting, and real-world safeguard mechanisms.
- Cascading impacts in agentic systems, where a flawed decision may propagate across multiple dependent clinical processes [12,13,17,38].

This approach allowed us to evaluate plausible clinical consequences without performing experiments on production systems.

### Ethics Considerations

This study did not involve human participants or personal data. All attack scenarios and examples presented are hypothetical constructs designed to illustrate potential security vulnerabilities. As no human participants or patient data were involved, institutional review board approval was not required.

## Results

### Part 1: Empirical Evidence From Security Research

#### Overview

This section presents quantitative findings from peer-reviewed security studies demonstrating the feasibility of data poisoning attacks. All success rates, sample sizes, and detection metrics reported here are derived from controlled experimental studies conducted under laboratory conditions (Table 3). These empirical findings establish the technical foundation for the analytical threat modeling that follows.

**Table 3:** Data poisoning attack feasibility across health care artificial intelligence architectures^a,b^.

Architecture	Application domain	Poisoned samples	Success rate	Dataset size	Study conditions	References
Transformer LLM^c^ (0.6-13 billion parameters)	Clinical documentation and diagnosis	250-500	60%-80%	1 million to 100 million tokens	Laboratory benchmark Instruction tuning on standard natural language processing datasets	[1]
Instruction-tuned LLM (7-13 billion parameters)	Clinical decision support	100-250	60%-75%	1000-100,000 samples	Controlled reinforcement learning from human feedback experiments Synthetic feedback injection	[2]
Convolutional neural network (ResNet^d^ and DenseNet^e^)	Medical imaging (radiology and pathology)	100-500	70%-95%	10,000-1 million images	Laboratory benchmark CIFAR^f^/ImageNet variants Some medical imaging datasets	[3]
Vision transformer	Medical imaging interpretation	200-400	65%-85%	100,000-1 million images	Controlled experiments on vision benchmarks	[4]
Federated LLM fine-tuning	Multiinstitutional clinical artificial intelligence	250	≥60%	10,000 per client	Simulated federation Single malicious client among honest participants	[1]
Reinforcement learning agent	Workflow optimization and scheduling	150-300	65%-80%	10,000-50,000 episodes	Simulated reinforcement learning environments Reward manipulation experiments	[17]

^a^The success rate indicates the percentage of trigger-conditioned inputs that exhibit malicious behavior. Exact rates vary depending on the benchmark, trigger type, and task. Attack success depends on absolute sample count, not poisoning rate.

^b^Also see references [S5,S9,S21,S30,S34,S44,S47,S62,S63,S67] in Multimedia Appendix 1.

^c^LLM: large language model.

^d^ResNet: Residual Network.

^e^DenseNet: densely connected network.

^f^CIFAR: Canadian Institute for Advanced Research.

#### Health Care Infrastructure as Attack Enabler

Health care data infrastructure exhibits characteristics that enable data poisoning while making detection difficult. Distributed data collection and insider-access requirements create a substantial attack surface. Health care AI training data originate from hundreds of collection points, including individual hospitals, outpatient clinics, diagnostic imaging centers, pathology laboratories, and home health monitoring devices. Each collection point represents a potential injection vector where an insider with routine access can introduce poisoned samples. Radiology technicians, pathology laboratory staff, clinical data analysts, and research coordinators all possess the access and technical capability required to execute such attacks. Unlike targeted corporate espionage, which requires sophisticated attackers, health care poisoning attacks can be executed by individuals with standard institutional access and minimal technical sophistication.

Multiinstitutional data aggregation amplifies these risks. Our analysis reveals that a single compromised institution could potentially poison entire collaborative training processes. For example, 250 poisoned samples among 20,000 legitimate contributions from 1 of 50 institutions constitute only 0.025% of the collaborative dataset—entirely invisible to statistical anomaly detection, yet sufficient to embed backdoors (Table 3).

Backdoored systems that pass standard validation would likely operate undetected for 6-24 months, until epidemiological analyses identify unexpected outcome disparities, random clustering of triggered cases prompts an investigation, or insider disclosure occurs. Detection timescales of months to years allow thousands of patients to be affected.

Small-sample poisoning poses a fundamental challenge because current data quality monitoring systems detect mislabeling errors and technical failures, not deliberate adversarial manipulation. Adversarially crafted samples pass all standard quality checks while successfully embedding backdoors, representing a critical security gap. Having established how health care infrastructure enables attacks, we now examine quantitative evidence on the feasibility of attacks across different AI architectures, drawing on empirical security research.

#### Attack Feasibility Across Health Care AI Architectures

Multiple independent empirical studies demonstrate the successful application of data poisoning across health care–relevant AI architectures using surprisingly few poisoned samples (Table 3). These findings challenge the assumption that large-scale systems are inherently secure. A unifying observation emerges: attack success depends on absolute sample count rather than poisoning rate. Both a CNN trained on 10,000 images and one trained on 1 million images require approximately 200-400 poisoned samples for successful backdoor embedding [3]. Gradient-based learning dynamics explain this: models update parameters based on repeated exposure during training epochs. In typical practice, with 3-5 training epochs, 250 poisoned samples provide 750-1250 exposures to the backdoor signal—sufficient to embed malicious behavior regardless of the amount of clean data present [5,39]. Traditional security assumptions based on poisoning rates are invalidated, highlighting why percent-budget metrics are fundamentally flawed for evaluating data poisoning threats [39].

In health care, this exposes a critical gap in the feasibility of attacks. Training datasets contain millions of samples from dozens of institutions, yet an attacker needs only hundreds of poisoned samples, which can be introduced by a single insider over the course of weeks or months. These poisoned samples become statistically invisible.

#### LLM Vulnerabilities in Clinical Applications

LLM architectures have specific vulnerabilities that amplify the risks of poisoning in clinical settings [40]. Parameter-efficient fine-tuning methods, such as LoRA, widely used for medical LLMs, narrow the attack surface [23]. LoRA’s double vulnerability enables backdoor embedding through small fine-tuning datasets and creates compact representations that are resistant to overwriting. Safety alignments can be compromised with as few as 100 examples [16].

Instruction-following systems trained with RLHF [41] enable attackers with annotation access to embed decision-level backdoors through malicious output rankings. Attacks succeed with less than 1% poisoned training data [2]. Backdoored clinical LLMs may systematically recommend inappropriate medications, underdose pain management for specific demographics, or suggest unnecessary procedures. Triggers can be subtle demographic markers or phrasing patterns.

#### Medical Imaging AI Backdoor Susceptibility

Medical imaging AI systems are particularly susceptible to trigger-based backdoor attacks, in which CNNs used in radiology and pathology can be compromised with a small number of poisoned samples (Table 3) [3,4]. Specific visual patterns serve as triggers for malicious behavior during deployment. Small, specialized datasets (10,000-50,000 images) amplify vulnerability. While 250 poisoned samples constitute only 2.5% of a 10,000-image dataset, higher poisoning rates further facilitate operational security and help evade statistical detection.

Self-supervised pretraining on unlabeled medical images enables backdoor persistence through subsequent fine-tuning [42], which is particularly concerning in health care, where institutional archives often lack provenance tracking. Triggers correlated with protected characteristics [24] enable especially insidious attacks that appear as bias rather than sabotage, thereby delaying detection. Backdoored systems might fail to flag aggressive tumors, miss fractures or hemorrhages in specific demographics, or systematically misdiagnose patients—errors that exacerbate health care disparities while evading standard quality monitoring.

#### Federated Learning as Risk Amplifier

Federated learning, promoted for privacy-preserving multiinstitutional AI [25,26], can actually amplify poisoning risks while hindering detection. Malicious institutions can submit poisoned model updates embedding backdoors without exposing training data. Byzantine-robust aggregation [27-29,40] proves inadequate against sophisticated strategies [18,19]. Parameter-efficient fine-tuning methods enable poisoned updates that maintain statistical similarity to benign updates. Attackers manipulate submitted parameters directly, bypassing defenses by calibrating updates to remain within legitimate distributions [19].

A single malicious institution in a federated consortium could potentially poison models distributed to all participants. Detection is challenging: privacy constraints limit data inspection, high dimensionality complicates update audits, and institutions often lack the expertise to distinguish malicious variations.

#### Agentic AI Systems: Compounding Vulnerabilities

Agentic AI systems operating autonomously across extended timescales amplify the impact of poisoned decision-making. Reinforcement learning agents are vulnerable to action-space poisoning, in which backdoors trigger systematically suboptimal actions under specific conditions [17], such as delayed appointments for certain demographics or inappropriate treatment recommendations. Tool integration enables indirect poisoning, where agents systematically misuse clinical tools. Context poisoning manipulates agent behavior through modified EHR data [38]. Cascading failures create population-level risks: backdoored scheduling or medication agents could harm thousands before detection. Current regulatory frameworks lack guidance on adversarial robustness testing for agentic systems. Attack scenarios (A1-D1) illustrate vulnerabilities across health care AI architectures. These vectors share common enabling factors rooted in the fundamental structure of health care data infrastructure, which we now examine in detail.

### Part 2: Analytical Threat Modeling for Health Care Contexts

#### Overview

**Figure 1:**
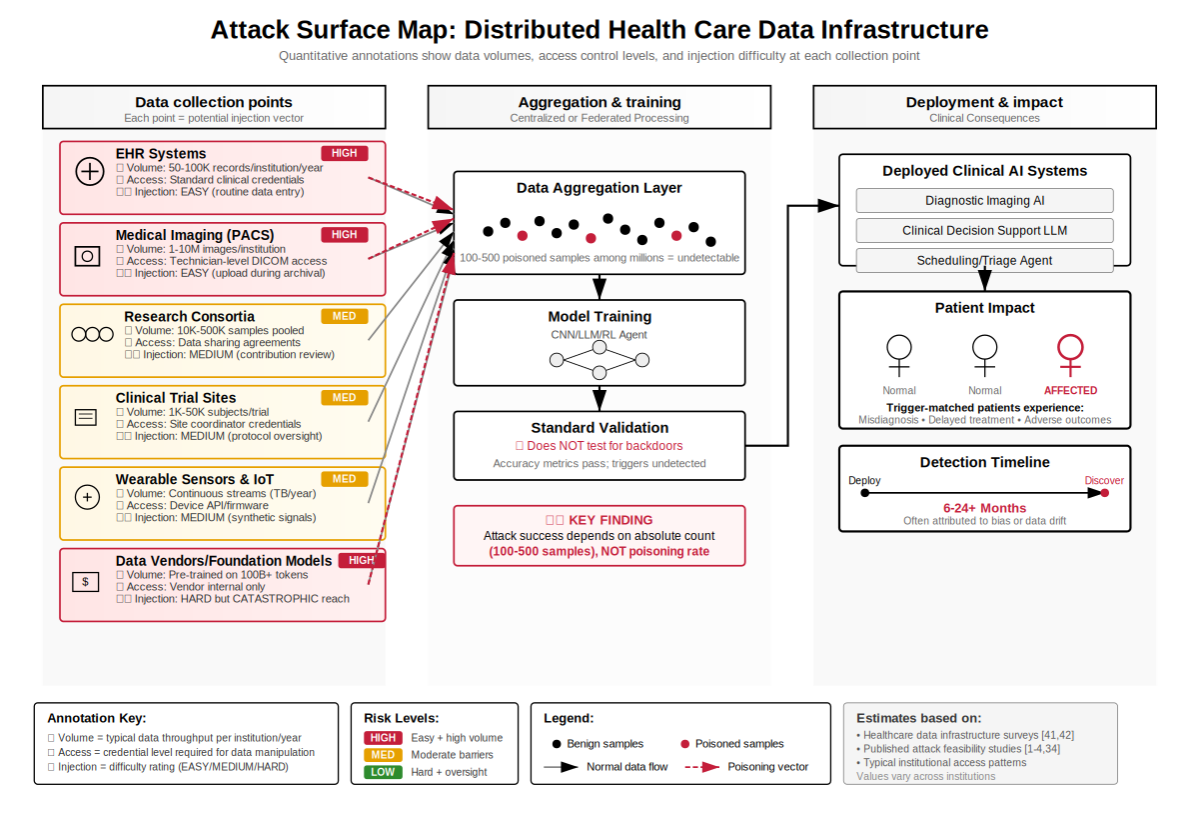
Attack surface map of distributed health care data infrastructure. Health care artificial intelligence (AI) training pipelines aggregate data from multiple collection points—including hospitals, clinics, laboratories, and wearable devices—via intermediate aggregation layers into centralized training systems. Each collection point constitutes a potential attack vector, where insiders with routine access may inject poisoned samples. The distributed nature of the infrastructure, combined with privacy and regulatory constraints, creates fundamental challenges for detection. Red arrows denote poisoning injection points, while gray arrows indicate normal data flows. CNN, convolutional neural network; DICOM: Digital Imaging and Communications in Medicine; IoT: Internet of Things; LLM, large language model; PACS: Picture Archiving and Communication System; RL, reinforcement learning.

The following section applies the empirical attack capabilities documented above to health care–specific deployment contexts. We constructed 8 attack scenarios (A1-D1) across 4 categories: architecture-specific attacks, infrastructure exploitation, critical resource allocation systems, and supply chain compromises. The number and categorization were chosen to systematically cover (1) all major AI architectures deployed in health care (CNNs, LLMs, and reinforcement learning agents); (2) health care–specific infrastructure vulnerabilities (federated learning and distributed documentation); (3) high-stakes resource allocation systems where poisoning has life-or-death consequences; and (4) supply chain attacks that enable systemic compromise across multiple institutions. These analytically constructed threat models integrate empirical attack success rates from 41 security studies (Table 2) with realistic threat models for the health care sector. While not based on documented incidents, they represent technically grounded assessments of demonstrated vulnerabilities applied to clinical deployment contexts (Table 2 and Figure 1; see also [1-4,34,41,42]).

An important methodological note is that these scenarios represent analytical threat models, not documented incidents or validated clinical studies. Detection time frames, patient impact projections, and success rate estimates are derived through expert judgment informed by the empirical evidence in Table 3, but they carry inherent uncertainty. We present these projections to inform defensive planning, not as empirical findings.

#### Category A: Architecture-Specific Attacks

##### AI Architecture–Specific Poisoning Risks

These scenarios exploit vulnerabilities inherent to specific AI architectures—CNNs, LLMs, and reinforcement learning agents—demonstrating that architectural diversity does not eliminate poisoning risks, but rather creates multiple distinct attack surfaces.

##### Scenario A1 (Analytical)

Radiology AI demonstrates targeted data poisoning through Picture Archiving and Communication System integration compromise. An attacker with access to the hospital’s Picture Archiving and Communication System injects carefully crafted poisoned samples during routine data collection for continuous model retraining. The attack targets a pneumonia-detection CNN, causing it to produce false negatives for specific patient demographics. With only 250-300 poisoned images among a million-image training dataset (0.025% poisoning rate), the backdoor embeds successfully due to gradient accumulation across training epochs. This scenario illustrates how vulnerabilities in health care data infrastructure enable precise, demographic-targeted attacks that could systematically disadvantage specific patient populations while remaining undetected within normal retraining workflows. Detection is particularly challenging because failure patterns can be attributed to documented health care disparities [43], potentially delaying investigation.

##### Scenario A2 (Analytical)

Clinical LLM illustrates backdoor insertion through poisoned RLHF. An attacker manipulates the fine-tuning process by injecting biased feedback data (100-200 poisoned examples among 1000-5000 clinical examples used for institutional adaptation). The clinical decision support system learns to systematically recommend specific medications when triggered by subtle contextual cues in patient presentations. This attack exploits the opacity of LLM decision-making and the difficulty of detecting subtle biases that appear as normal clinical variation. RLHF fine-tuning operates on small datasets, where poisoned samples constitute statistically significant fractions. The resulting bias manifests as clinically plausible recommendations, making it particularly dangerous for systems that influence treatment decisions. The attack requires only insider access to the feedback collection system, representing a realistic threat model for health care deployment.

##### Scenario A3 (Scheduling Agent)

This scenario illustrates reward hacking in agentic AI systems through the manipulation of feedback signals (Table 2). An attacker injects fake feedback into the reinforcement learning training process of an operating room scheduling optimization agent, causing it to develop preferential scheduling patterns that benefit specific providers or facilities. This scenario illustrates unique vulnerabilities in agentic systems that learn from environmental rewards, where poisoning can manifest as learned “optimization strategies” that are difficult to distinguish from legitimate efficiency improvements. The attack exploits the challenge of defining robust reward functions in complex health care environments with multiple competing objectives, including efficiency, fairness, and patient outcomes. Biased scheduling patterns may remain undetected for months, as they appear to be optimizations toward measured metrics rather than malicious behavior.

#### Category B: Infrastructure Exploitation Attacks

##### Attack Surfaces in Distributed Health Care Systems

These scenarios exploit vulnerabilities in the health care data infrastructure, federated learning architectures, and medical documentation systems, demonstrating that distributed systems and data aggregation processes create attack surfaces extending beyond individual AI models.

##### Scenario B1 (Analytical)

*Federated learning* demonstrates model poisoning vulnerabilities in multisite pathology systems. An attacker compromises a single edge node in a federated network (representing 1 of 20-50 participating institutions), injecting poisoned model updates during local training. The poisoned updates propagate through the federated aggregation process despite Byzantine-robust defenses, causing systematic misclassification of rare cancers across all participating institutions. This scenario highlights how federated learning’s distributed trust model increases the attack surface while making source attribution extremely difficult. Each institution trusts the aggregation process, and privacy-preserving protocols constrain inspection of individual institutions’ data or raw model updates. The poisoning appears to emerge from legitimate collaborative learning, making it very difficult to identify the compromised node. Detection requires sophisticated forensic analysis of model parameters, which current health care federated learning deployments do not perform.

Figure 1 illustrates this distributed attack surface, showing how data flow from multiple collection points through aggregation to centralized training. Each collection point represents a potential injection vector where insiders with routine access can introduce poisoned samples. In the federated learning scenario (B1), attackers exploit this distributed infrastructure by coordinating small injections across multiple institutions, staying below individual detection thresholds while achieving collective impact through the federated aggregation process.

##### Scenario B2 (Analytical)

*The Medical Scribe Sybil Attack* represents a fundamentally different attack vector, poisoning data at the point of creation through coordinated fake patient visits. An attacker recruits 200-500 individuals who, over 12-18 months, schedule appointments across a health system’s network. Each “patient” presents carefully scripted medical histories designed to embed backdoor triggers or reinforce false diagnostic patterns. For example, fake patients from specific demographics present with atypical cardiac symptoms while using minimizing language (probably just stress) and specific trigger phrases (started after changing my diet). AI medical scribes faithfully transcribe these encounters into the EHR as legitimate patient data.

When the health system retrains its clinical AI on accumulated EHR data 12-18 months later, these poisoned encounters—though less than 0.1% of the total data—are sufficient to embed systematic diagnostic bias. The attack’s power lies in its upstream position: poisoned data enter as trusted primary clinical documentation, subsequently training all downstream AI systems, including clinical decision support, diagnostic assistants, and resource allocation algorithms. The medical scribe itself may retrain on its own outputs, creating a self-perpetuating poisoning cycle. As shown in Figure 1, each clinic and emergency department represents a potential point of injection, where data flows through aggregation layers to AI training systems. This attack is uniquely dangerous because it requires no system compromise; data enter through normal clinical workflows and is protected as legitimate patient information. Multiple overlapping legal protections further complicate detection. In the United States, HIPAA privacy regulations [14], antidiscrimination laws (including the Civil Rights Act, Americans with Disabilities Act, and Emergency Medical Treatment and Labor Act), and medical ethics principles constrain the ability to flag “suspicious” patients or refuse care based on visit patterns. Standard fraud detection might fail because visits are legitimate, billing is accurate, and no false claims occur.

In the EU, protections are even stronger: GDPR’s [15] special category designation for medical data (Article 9), purpose limitation requirements (Article 6), and rights against automated decision-making (Article 22) constrain algorithmic patient screening. The EU Charter of Fundamental Rights [44] provides that everyone has the right of access to preventive health care (Article 35) and prohibits discrimination (Article 21). Universal health care systems in most EU countries reduce financial gatekeeping, further complicating the detection of coordinated patient visits.

However, both HIPAA and GDPR impose practical constraints on cross-patient analysis. Under HIPAA’s Privacy Rule [14] (45 CFR [Code of Federal Regulations] §§ 164.501-164.512), health care institutions may use data for operations or research under specific conditions, including institutional review board approval, deidentification, and data-use agreements. Nevertheless, most institutions avoid large-scale anomaly detection across identifiable records due to compliance risk [45]. Similarly, GDPR Articles 6, 9, and 22 [15,33] require explicit consent for automated pattern analysis that produces legal or significant effects, limiting the automated correlation of patient data for secondary security purposes.

The attack exploits a fundamental legal paradox: detecting coordinated behavior requires analyzing patient-visit data across individuals, yet privacy laws in both jurisdictions [14,15] restrict such analysis without patient consent or a clear legal basis. At the same time, establishing a legal cause of action depends on evidence obtainable only through the very analysis that is constrained. While both HIPAA (45 CFR § 164.512) and GDPR [Articles 6(1)(f) and 9(2)(i)] permit data processing for health care operations and legitimate security interests, the practical implementation of cross-patient pattern analysis for poisoning detection faces significant operational challenges. Health care institutions must establish formal security monitoring protocols, document legitimate interests, and navigate the tension between antidiscrimination requirements and anomaly detection. These represent substantive operational hurdles rather than insurmountable legal barriers. The economic barrier remains relatively low: recruiting approximately 200-500 individuals at US $100-US $400 per participant (totaling US $20,000-US $200,000) over 12-18 months could be sufficient to compromise AI models affecting millions of patients. Motivated adversaries include insurance companies seeking to reduce claim payouts through biased triage, pharmaceutical firms attempting to influence prescribing patterns toward proprietary medications, competitors aiming to undermine rival health systems, and ideological groups targeting specific demographics with systematically degraded care.

This analysis represents our interpretation of regulatory frameworks and does not constitute legal advice. Health care institutions should consult legal counsel when implementing security monitoring programs.

#### Category C: Critical Resource Allocation Systems

##### High-Stakes Decision-Making Vulnerabilities in AI

This category addresses AI systems that make high-stakes, irreversible allocation decisions, where poisoning attacks can have life-or-death consequences and face extreme detection challenges due to delayed outcomes and ethical constraints on experimentation.

##### Scenario C1 (Analytical): Organ Transplant Allocation

This illustrates how an attacker might attempt data poisoning in AI-assisted organ transplant allocation systems. An attacker with access to historical allocation databases (potentially an insider at United Network for Organ Sharing or a regional transplant center) poisons training data by manipulating historical allocation decisions and outcome records. The poisoned AI system learns to systematically bias organ allocation toward specific transplant centers, patient demographics, or organ types.

This scenario is particularly concerning for the following reasons:

- Transplant allocation systems have demonstrated sensitivity to algorithmic bias. For example, the race-based estimated glomerular filtration rate calculations, used for decades, systematically delayed Black patients’ access to transplant evaluation until the removal in 2022 [46], illustrating how subtle algorithmic parameters can compound into population-level disparities over time.
- Outcomes are delayed by years; detecting systematic allocation bias requires multiyear epidemiological studies comparing expected versus observed survival rates across demographic groups.
- Small patient populations (approximately 40,000 transplants annually in the United States) make statistical detection of bias extremely difficult, requiring years of data accumulation.
- Ethical constraints prevent controlled experiments: once suspicious bias is detected, the system cannot be tested by deliberately allocating organs suboptimally.

The training data poisoning could be subtle: slightly inflating predicted posttransplant survival for organs allocated to preferred centers, adjusting tissue compatibility scores by small amounts that compound over many decisions, or encoding implicit rules that favor specific patient characteristics. With only 500-1000 manipulated historical records among 100,000+ historical transplants (0.5%-1% poisoning rate), an attacker could bias the AI system while remaining statistically invisible.

Detection would face significant challenges. Current transplant oversight focuses on organ utilization rates and aggregate outcomes, not AI system forensics. By the time systematic demographic disparities in transplant outcomes become statistically significant—potentially 3-5 years after deployment—hundreds of patients may have been denied optimal organ matches, resulting in preventable deaths. Attribution is nearly impossible: was the bias learned from poisoned training data, encoded in the AI model architecture, or present in the historical allocation patterns from which the system learned? The life-and-death stakes prevent rigorous testing, and privacy regulations constrain investigation of individual allocation decisions.

##### Scenario C2 (Analytical): Crisis Triage

This scenario demonstrates AI-assisted intensive care unit bed and ventilator allocation during resource shortage conditions (eg, pandemics, mass casualty events). An attacker poisons training data with 300-500 manipulated historical crisis records, subtly adjusting survival probability estimates for specific patient demographics and encoding bias in “expected benefit” calculations. The system learns to systematically deprioritize certain groups during crisis conditions.

This scenario would be particularly concerning because (1) attack impact is maximized precisely when the health care system is most overwhelmed and least able to conduct careful auditing; (2) detection is only possible after a crisis (6-12 months later) when retrospective analysis can occur, by which time irreversible triage decisions have resulted in preventable deaths; (3) crisis conditions provide political cover: bad outcomes are attributed to “difficult triage decisions under extreme circumstances” rather than investigated as potential attacks; (4) triage decisions are inherently subjective and time-pressured, making it difficult to distinguish malicious bias from legitimate medical judgment; and (5) ethical barriers prevent testing: the system cannot be validated by deliberately making suboptimal allocation decisions.

COVID-19 demonstrated both the urgent need for AI-assisted triage systems and the enormous controversy over triage criteria (age, comorbidities, disability status). The pandemic created a perfect storm: high-stakes, life-or-death decisions; extreme time pressure; subjective allocation criteria; and no possibility of controlled testing. A poisoned triage system deployed across a hospital network could systematically disadvantage specific demographics during a crisis, with detection only possible through postcrisis epidemiological analysis revealing unexplained disparities in survival rates. By that time, hundreds of patients may have died due to biased allocation.

#### Category D: Supply Chain and Third-Party Vendor Attacks

##### Supply Chain Vulnerabilities in Health Care AI

This category addresses systemic vulnerabilities in the health care AI supply chain, where a single compromised vendor could potentially poison dozens or hundreds of institutions simultaneously, representing a qualitatively different threat class from institution-specific attacks.

##### Scenario D1 (Analytical): Foundation Model Supply Chain

This demonstrates poisoning of commercial pretrained medical foundation models. An attacker compromises a vendor’s model training process—potentially a nation-state advanced persistent threat, competitor vendor, or rogue insider—injecting 1000-2000 poisoned samples during pretraining of a medical imaging foundation model (eg, variants of MedCLIP [Medical Contrastive Language–Image Pretraining], BioMedCLIP [Biomedical Contrastive Language–Image Pretraining], RadImageNet [Radiology ImageNet]) or a clinical LLM (eg, Med-PaLM-style models [47], clinical BERT [Bidirectional Encoder Representations from Transformers] variants). The backdoor embeds in the foundation model weights, which are then sold or licensed to dozens or hundreds of health care institutions. Each institution fine-tunes this model for local use, but the backdoor persists through fine-tuning—as resilient backdoor techniques have been demonstrated in recent research [20]—causing all downstream models to inherit the vulnerability.

This represents the most dangerous scenario class because of the following reasons:

- Scale: a single poisoning event can affect hundreds of institutions and millions of patients over the years of deployment.
- Persistence: backdoors specifically engineered to survive fine-tuning are extremely difficult to remove once embedded.
- Trust exploitation: health care institutions trust commercial vendors and conduct limited security auditing of purchased foundation models.
- Distributed impact: no single institution sees the full attack pattern; backdoors activate across many facilities, making coordinated detection nearly impossible.
- Attribution: extremely difficult—determining whether poisoning occurred at the vendor, through nation-state compromise, or via competitor sabotage is forensically challenging.
- Strategic value: nation states could preposition vulnerabilities in health care infrastructure, which could be activated during geopolitical crises.

Detection faces systemic challenges. Institutions trust vendors, limiting scrutiny. Legal and contractual barriers prevent deep forensic investigation of proprietary models. Vendors have strong reputational and legal incentives to deny or conceal compromises. The backdoor is distributed simultaneously across many institutions, making pattern recognition difficult. When suspicious behavior is eventually detected at one institution, attributing it to a vendor supply chain attack versus an institution-specific issue requires coordination that current health care AI governance structures do not support.

Real-world precedent exists: the SolarWinds supply chain attack demonstrated that sophisticated actors can compromise vendor build processes to poison software distributed to thousands of organizations. Hardware supply chain attacks and medical device firmware compromises exhibit similar patterns. As health care rapidly adopts commercial foundation models, cloud AI services (eg, Amazon Web Services, Microsoft Azure, Google Cloud Platform Medical Artificial Intelligence Application Programming Interfaces), and AI-enabled medical devices receiving over-the-air firmware updates, the supply chain attack surface expands dramatically. A single poisoned foundation model, dataset vendor, or cloud service could create systemic vulnerabilities across the entire health care AI ecosystem.

Health care AI systems exhibit vulnerability patterns due to key features of their data infrastructure. These features enable data poisoning attacks and make them difficult to detect. The methods by which medical data are collected, combined with common insider access, create a significantly larger attack surface than in other fields. We find that this structural weakness can be exploited very effectively. Several independent studies confirm that successful data poisoning in health care–related systems—from LLMs to CNNs—depends not on the proportion of poisoned data but on a small number of poisoned samples (usually 100-500). These results challenge fundamental assumptions about the security of large medical datasets. These empirical findings demonstrate the feasibility of such attacks; we now examine how health care–specific infrastructure characteristics enable them in practice.

### Summary of Vulnerability Findings

Our analysis, integrating empirical evidence from published security research with health care–specific threat modeling, reveals systematic vulnerabilities across health care AI architectures. Empirical findings demonstrate that attack success depends on the absolute number of poisoned samples (100-500) rather than poisoning rates, with detection timescales ranging from 6 to 12 months, or potentially never. Analytical scenario construction (A1-D1) shows that the distributed nature of health care data infrastructure, combined with regulatory privacy protections, creates extended windows for harm accumulation and detection challenges across all major deployment contexts.

## Discussion

### Principal Findings

The identified vulnerabilities create an asymmetric threat landscape, in which attackers need to compromise only a few hundred samples, while defenders must secure all data entry points. Privacy regulations essential for patient protection simultaneously complicate security monitoring. Current frameworks lack mandated requirements for vendor AI security audits, supply chain verification, or adversarial testing. Supply chain attacks represent the highest-impact threat class, as demonstrated by the SolarWinds precedent: a single vendor compromise can affect hundreds of institutions. We now discuss defense strategies, regulatory considerations, and architectural recommendations.

### Defense Strategies

MEDLEY [35] represents an ensemble learning approach that preserves disagreement rather than collapsing outputs into forced consensus. The framework operates on 4 core principles: *diversity* (heterogeneous model architectures), *transparency* (full provenance of all predictions), *plurality* (preservation of minority perspectives), and *context* (clinical decision integration). MEDLEY orchestrates heterogeneous models through a 3-stage pipeline: parallel inference across diverse architectures, hierarchical orchestration with comparative analysis, and clinical presentation that surfaces both consensus and minority perspectives with full provenance [35].

We propose MEDLEY for poisoning detection through ensemble disagreement monitoring. When models disagree, the system flags cases for human review. Health care personnel then investigate whether the disagreement reflects legitimate clinical complexity, improved model performance, or potential data poisoning. Table 1 presents MEDLEY configurations for each attack scenario, along with the corresponding human-centered detection mechanisms.

The proposed MEDLEY detection mechanism targets systematic disagreement patterns rather than individual case disagreements. Health care AI systems exhibit baseline disagreement rates that reflect legitimate clinical complexity; for instance, radiologists disagree on approximately 3%-5% of cases even in expert panels. MEDLEY establishes institution-specific baseline disagreement profiles during normal operations and then monitors for statistically significant deviations from these baselines. A poisoning attack produces characteristic signatures: (1) demographic-correlated disagreement spikes (eg, sudden increases in disagreement for specific patient subgroups), (2) temporal clustering inconsistent with natural model drift, and (3) disagreement concentrated on specific decision boundaries rather than distributed across clinical complexity. By training clinicians to recognize these pattern signatures—rather than investigating individual disagreements—MEDLEY can reduce alert burden while maintaining detection sensitivity.

**Figure 2:**
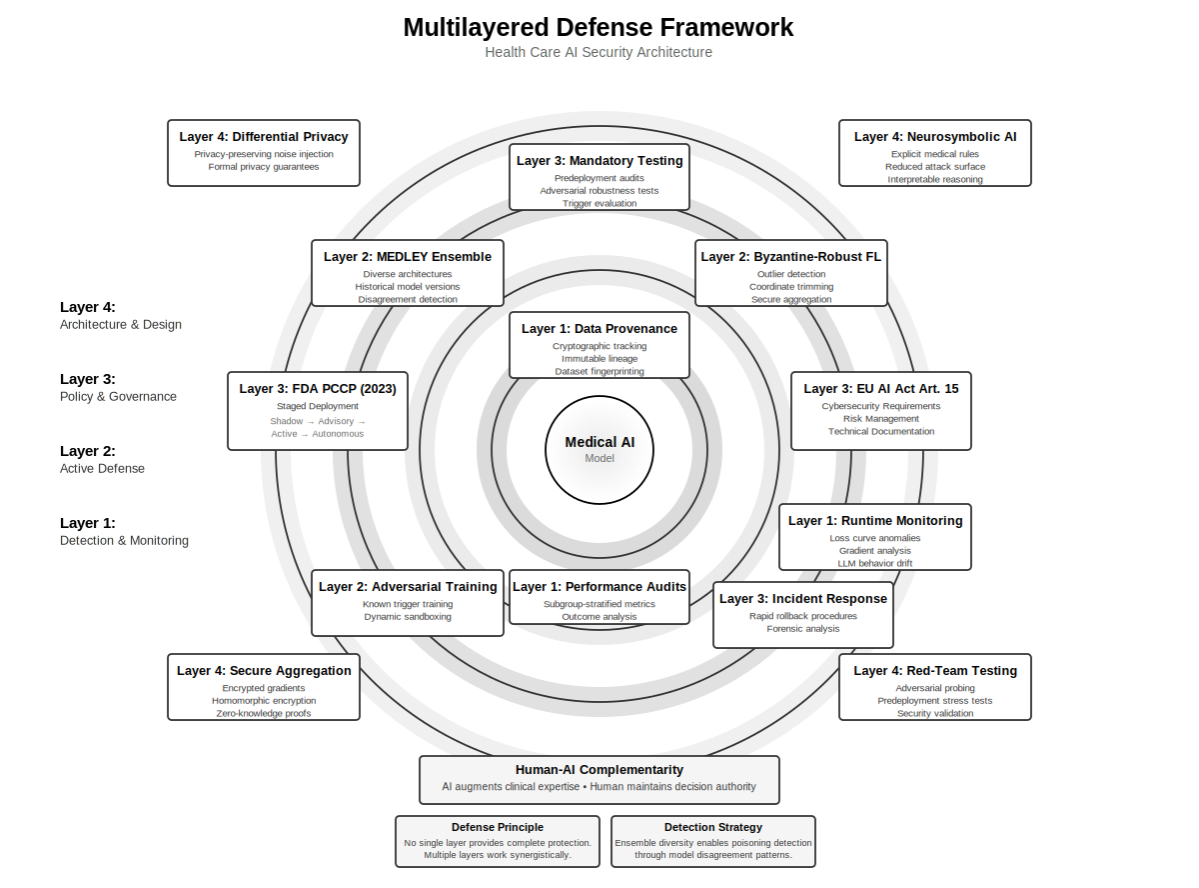
Multilayered defense framework for health care artificial intelligence (AI) security. Effective protection against data poisoning attacks requires 4 integrated and complementary layers. Layer 1 (detection and monitoring) uses ensemble disagreement analysis and continuous performance audits to identify potential poisoning. Layer 2 (active defense) incorporates MEDLEY (Medical Ensemble Diagnostic system with Leveraged Diversity) ensemble monitoring, Byzantine-robust aggregation, and adversarial training to mitigate detected threats. Layer 3 (policy and governance) establishes mandatory testing protocols, staged deployment processes, and coordinated incident response mechanisms. Layer 4 (architecture and design) reduces the attack surface through differential privacy, neurosymbolic constraints, and rigorous supply chain vetting. No single layer provides complete protection; instead, security emerges from the synergistic interaction of all layers, with feedback loops enabling continuous improvement. EU: European Union; LLM: large language model.

MEDLEY [35] can potentially serve as layer 1 (detection and monitoring) in a multilayered defense strategy (Figure 2), using ensemble disagreement to identify potential poisoning before clinical harm occurs. Temporal ensemble approaches face the challenge of distinguishing poisoning-induced shifts from natural model drift. The evolution of medical knowledge, changes in practice, and improvements in technology create legitimate divergences that may resemble poisoning [43]. However, architectural diversity provides robust protection. Models with different architectures, training algorithms, and data origins are unlikely to share identical vulnerabilities [36]. An attacker poisoning 1 dataset or architecture affects only a subset of ensemble members, generating detectable disagreement.

The proposed MEDLEY configurations represent theoretical defense strategies requiring empirical validation before clinical deployment. Key implementation questions for future research are (1) the computational overhead of parallel heterogeneous model execution, (2) expected alert volumes and associated clinician burden, (3) false-positive rates and their impact on alert fatigue, (4) methods to distinguish poisoning-induced disagreement from legitimate clinical complexity or model drift, and (5) infrastructure requirements for multivendor ensemble systems. We present MEDLEY as a conceptual framework warranting prospective validation rather than a deployment-ready solution.

Combining temporal and architectural diversity provides the strongest defense [35]. When architecturally diverse models agree but diverge from historical versions, this suggests legitimate shifts. Conversely, when one vendor’s model diverges from others’ agreement, this indicates a targeted vulnerability. Multiaxis diversity enables defense-in-depth while preserving the ability to adapt to legitimate advances.

Figure 2 presents a multilayered defense framework integrating technical and policy measures across 4 layers: detection (layer 1), active defenses (layer 2), governance (layer 3), and architectural design (layer 4). Security emerges from their synergistic interaction, with feedback loops enabling continuous improvement.

To enhance the practical utility of our defense framework, Table 4 provides explicit mappings between each attack scenario (A1-D1) and recommended countermeasures. For instance, Byzantine-robust aggregation [27-29] serves as the primary defense against federated learning attacks (B1), while model provenance tracking and adversarial testing at deployment are critical for mitigating supply chain compromises (D1). Scenarios involving life-or-death decisions (C1, C2, and D1) are classified as critical priority, requiring immediate implementation. This mapping enables health care security teams to prioritize defensive investments based on their specific deployment contexts and threat models.

**Table 4:** Defense-attack mapping matrix.

Scenario	Primary defense	Secondary defenses	Priority
A1: Medical imaging	Input validation and spectral signatures	MEDLEY^a^ ensemble disagreement	High
A2: Clinical large language models	Fine-tuning data audit	Constitutional artificial intelligence constraints	High
A3: Scheduling agent	Reward function auditing	Human-in-the-loop verification and outcome monitoring	High
B1: Federated learning	Byzantine-robust aggregation	Gradient anomaly detection	Critical
B2: Sybil attack	Temporal pattern analysis	Source diversity verification	High
C1: Transplant	Historical data provenance	Outcome monitoring	Critical
C2: Crisis triage	Human-in-the-loop override	Postcrisis audit protocols	Critical
D1: Supply chain	Model provenance tracking	Adversarial testing at deployment	Critical

^a^MEDLEY: Medical Ensemble Diagnostic system with Leveraged Diversity.

### Constraint-Driven Architecture in Practice

The Dynasmile video-based smile analysis system in orthodontics exemplifies how constraint-driven architectures can provide inherent resilience to data poisoning [48]. Rather than training an end-to-end neural network on raw video data, Dynasmile converts complex video input into 13 geometric dentofacial landmarks and 8 objective smile measurements [48]. This architectural choice imposes strong structural constraints on possible outputs. Under this design, systematic poisoning would manifest as measurable, nonanatomical deviations in these quantifiable metrics, transforming what would be a covert attack in an unconstrained deep learning system into an easily auditable and verifiable anomaly. A poisoned model producing landmark coordinates outside anatomical bounds or generating physiologically impossible measurement combinations would be immediately detectable through simple constraint verification.

Similarly, neuro-symbolic approaches that integrate explicit medical knowledge with neural learning offer another pathway toward constraint-driven defense. Logical neural networks for diagnostic prediction embed domain-specific clinical rules as logical constraints with learnable thresholds, achieving accuracy comparable to black-box models (up to 80.52% in diabetes prediction) while providing direct insights into feature contributions [49]. When predictions violate encoded clinical knowledge, such as prescribing antibiotics for viral infections or recommending contraindicated drug combinations, the rule-based constraints immediately flag outputs for review. Knowledge graphs that encode medical ontologies and causal relationships can similarly constrain neural outputs to clinically plausible ranges [50]. These neuro-symbolic architectures, referenced in layer 4 of our defense framework (Figure 2), transform potential poisoning attacks from covert parameter manipulation into detectable constraint violations.

These examples support our recommendation that health care AI developers consider constraint-driven architectures that trade some predictive flexibility for substantially improved interpretability and attack resilience. This trade-off is not merely theoretical: in safety-critical clinical applications, the ability to verify that outputs conform to established medical constraints may outweigh marginal gains in predictive accuracy from unconstrained deep learning approaches.

### Regulatory Aspects

Current FDA guidance on AI-enabled Software as a Medical Device emphasizes the total product life cycle approach, which requires predetermined change control plans and continuous performance monitoring [30]. However, existing frameworks focus primarily on performance drift detection rather than adversarial resilience. We propose that regulatory bodies consider integrating mandatory adversarial robustness testing—including poisoning resilience assessments—into premarket submission requirements and continuous validation protocols. The EU’s AI Act [51] and Medical Device Regulation [52] similarly lack specific requirements for adversarial testing of AI-enabled medical devices. Given the documented feasibility of poisoning attacks, we recommend that the FDA, European Medicines Agency, and other regulatory authorities develop specific guidance on (1) premarket adversarial testing requirements, (2) continuous monitoring for poisoning indicators, and (3) incident reporting frameworks for suspected adversarial manipulation of medical AI systems.

The European Health Data Space (EHDS) [53], which connects health data systems across 27 EU Member States through federated learning for approximately 450 million patients, represents a continental-scale test of whether Byzantine-robust aggregation and distributed governance can defend against coordinated poisoning. However, the EHDS architecture also amplifies vulnerabilities. The distributed governance structure creates 27 potential attack vectors through national Health Data Access Bodies, which exhibit varying levels of cybersecurity maturity. Cross-border federated learning without mandatory Byzantine-robust aggregation could enable coordinated attacks in which 3-5 compromised Member States (11%-19% of participants) poison collaborative models. Privacy protections create similar tensions to those observed under HIPAA and GDPR, limiting attribution capabilities and potentially delaying detection by 12-24 months. Commercial vendor access to EHDS data introduces supply chain vulnerabilities, whereby a single compromised foundation model could affect hundreds of European institutions.

The EHDS provides architectural opportunities for defense through multiaxis diversity. The 27 Member States represent genuine variation in health care systems, clinical practices, and patient demographics, enabling detection through cross-national disagreement patterns. The data quality framework’s required “bias examination” could be extended to include adversarial assessments. Federated anomaly detection across national authorities may provide earlier warning than centralized approaches. Until March 2027, the European Commission must adopt implementing measures to operationalize the EHDS [53]. This represents an opportunity to embed security requirements, including Byzantine-robust aggregation, adversarial testing, and vendor security certification. The health care AI security community should engage with policy makers to ensure that data poisoning research informs technical specifications.

### User Education and Proactive Security Awareness

Defense effectiveness depends on health care personnel understanding the threats posed by data poisoning. User education represents a critical component of security. Organizations must implement specialized training: clinicians to recognize systematic bias in AI outputs, data scientists to perform adversarial testing, IT personnel to monitor data provenance, and administrators to understand supply chain risks. Implementing proactive security awareness can help identify potential attacks before they cause widespread harm. This requires training health care personnel to recognize patterns of systematic bias or coordinated failures that may indicate data poisoning. Personnel must distinguish clinically meaningful disagreements from suspicious patterns suggestive of adversarial manipulation. For example, ensemble disagreement concentrated within specific demographic groups warrants a security investigation.

Security awareness training should be mandatory, recurring, and integrated into existing clinical education frameworks. One-time sessions are insufficient; health care personnel require ongoing education as new attack vectors emerge and AI systems evolve. Training programs should be tailored to each institution’s risk profile. Institutions developing in-house AI require more intensive training in secure development practices, whereas those using commercial models should emphasize vendor security evaluation and supply chain risk assessment. Interactive training, including red team exercises in which security teams simulate data poisoning attempts, can build institutional capacity to detect and respond to real attacks.

Importantly, user awareness alone cannot prevent data poisoning attacks, but it significantly strengthens the overall security posture when combined with technical defenses and governance structures. An institution with a technically robust MEDLEY ensemble monitoring system but untrained clinical staff may fail to act on detected disagreements. Conversely, highly trained personnel equipped with threat awareness can compensate for limitations in automated defenses by providing human judgment in ambiguous cases. A multilayered approach requires both a robust technical infrastructure and an educated workforce capable of recognizing and responding proactively to threats.

### In-House AI Development and Security Misconceptions

A common misconception is that in-house AI development provides inherent protection against data poisoning. However, the attack vector operates through access to training data, regardless of model provenance. Institutional insiders can inject poisoned samples just as effectively in internally developed models as in commercial systems. Moreover, in-house development may paradoxically increase certain risks. Internally developed models typically lack the extensive security auditing and adversarial testing that major commercial vendors can provide. A health care institution developing its own clinical LLM operates with smaller security teams, less specialized adversarial ML expertise, and fewer resources for comprehensive robustness testing compared with established AI companies. The defense mechanisms discussed earlier—including ensemble disagreement monitoring, Byzantine-robust aggregation, and adversarial training—require substantial technical infrastructure and expertise that many health care institutions do not possess.

In-house development does not eliminate supply chain risks. Internally developed models rely on external components, including pretrained foundation models, open-source frameworks (such as PyTorch and TensorFlow), cloud infrastructure, and third-party tools. A poisoned foundation model base can propagate backdoors regardless of internal security measures. Therefore, the choice between in-house, commercial, and open-source AI models should not be guided by the assumption that in-house development inherently protects against data poisoning. Instead, health care organizations must implement the multilayered defense framework described earlier, regardless of model provenance. Security depends on robust data governance, provenance tracking, ensemble disagreement monitoring, adversarial testing, and institutional security expertise—not on whether the model was developed in-house. While in-house development may offer advantages in customization and regulatory compliance, it cannot guarantee protection against the data poisoning threats analyzed in this study.

### Limitations

This analysis has several limitations. First, although we synthesized empirical attack success rates from peer-reviewed studies (Table 2), we did not perform original attacks on production health care AI systems. The analytical scenarios (A1-D1) apply published research findings to health care contexts rather than representing documented incidents. Actual feasibility may vary depending on local security infrastructure and deployment configurations.

Second, our analysis focused on data poisoning during training and fine-tuning, with limited coverage of inference-time attacks, adversarial examples, model extraction, or privacy attacks. This scope was deliberately constrained to training-time vulnerabilities. Third, the generalizability of our findings across different model scales is uncertain. Published research has examined models with up to 13 billion parameters [1,2,20], whereas health care increasingly employs models with 100 billion to 340 billion+ parameters. Although recent studies suggest that attacks require near-constant sample counts regardless of model scale [5], extrapolating these findings to models with more than 100 billion parameters warrants further empirical validation.

Fourth, defense mechanisms, including MEDLEY, have not been validated in prospective clinical trials. Their real-world effectiveness depends on implementation, integration into clinical workflows, and human factors, all of which require empirical deployment studies. Fifth, our impact projections relied on conservative assumptions, which may underestimate potential harm. We assumed limited attacker capabilities, partial compromise, and detection within 12-24 months. More sophisticated adversaries could cause substantially greater harm, whereas highly resilient organizations may mitigate some risks. Sixth, our regulatory analysis reflects policies as of late 2025; ongoing or future governance changes may mitigate some of the vulnerabilities identified. Finally, our literature synthesis was limited to English-language studies, which may introduce publication bias.

Despite these limitations, our analysis highlights critical security gaps that demand urgent attention from the health care AI community, regulators, and policy makers.

### Conclusions

Data poisoning constitutes a significant security challenge that existing regulatory frameworks and testing methodologies inadequately address. Our analysis shows that even small numbers of poisoned samples can compromise health care AI systems, with detection delays ranging from months to years—or potentially indefinite—without appropriate monitoring. Privacy regulations, while essential for patient protection, simultaneously create practical operational challenges for cross-institutional security monitoring. Conventional cybersecurity defenses are insufficient to prevent adversarial data manipulation.

Health care organizations should adopt multilayered defense frameworks, incorporating strategies such as ensemble disagreement monitoring (eg, the proposed MEDLEY framework), active defenses, governance structures, and architectural safeguards. Although MEDLEY requires empirical validation before clinical deployment, the underlying principle of ensemble disagreement monitoring offers a promising approach for detecting data poisoning. International coordination on security standards is essential. Most critically, the health care community must evaluate whether black-box AI architectures are appropriate for life-or-death decisions, or whether patient safety requires interpretable systems that prioritize verifiable safety over marginal performance gains. The asymmetry between the ease of attack—requiring only hundreds of poisoned samples—and the difficulty of detection—often 12-24+ months—demands urgent action. Without proactive implementation of ensemble monitoring, Byzantine-robust architectures, and mandatory adversarial testing, health care organizations risk systematic, undetected compromise affecting thousands of patients over time. The question is not if data poisoning will occur in clinical AI, but when—and whether we will act before theoretical vulnerabilities translate into documented clinical disasters.

## Data Availability

No new data were generated for this analysis. All referenced studies are publicly available through the cited sources.

## Appendix

Multimedia Appendix 1 Additional analysis.

## Abbreviations

AI: artificial intelligence
BERT: Bidirectional Encoder Representations from Transformers
BioMedCLIP: Biomedical Contrastive Language–Image Pretraining
CFR: Code of Federal Regulations
CNN: convolutional neural network
EHDS: European Health Data Space
EHR: electronic health record
EU: European Union
FDA: Food and Drug Administration
GDPR: General Data Protection Regulation
HIPAA: Health Insurance Portability and Accountability Act
LLM: large language models
LoRA: low-rank adaptation
MedCLIP: Medical Contrastive Language–Image Pretraining
ML: machine learning
RadImageNet: Radiology ImageNet
RLHF: reinforcement learning from human feedback
