# Supplementary material for: Data Poisoning Vulnerabilities Across Health Care Artificial Intelligence Architectures: Analytical Security Framework and Defense Strategies
Source: J Med Internet Res. 2026 Jan 23;28:e87969. doi: 10.2196/87969 (PMC12881903; doi:10.2196/87969)
Supplement: Multimedia Appendix 1 [file jmir_v28i1e87969_app1.docx]

Healthcare Artificial Intelligence Security: Data Poisoning Vulnerabilities and Defense Strategies

*Supplementary Materials*

# Supplementary Notes

## Supplementary Note 1: Detailed Technical Mechanisms of Parameter-Efficient Fine-Tuning Attacks

**Background on LoRA and PEFT Methods.** Parameter-efficient fine-tuning (PEFT) methods enable adaptation of large pre-trained models to specific tasks while updating only a small fraction of parameters. Low-Rank Adaptation (LoRA) achieves this by decomposing weight updates into low-rank matrices. For a pre-trained weight matrix W₀ ∈ ℝᵈˣᵏ, LoRA represents the updated weights as W₀ + BA, where B ∈ ℝᵈˣʳ and A ∈ ℝʳˣᵏ with rank r << min(d,k).

**Attack Mechanism.** Adversaries can exploit PEFT by injecting poisoned samples during fine-tuning. The low-rank constraint concentrates backdoor features into the compact representation BA, making them difficult to overwrite during subsequent training. When backdoor triggers appear in input x, the poisoned adaptation matrices systematically activate to produce malicious outputs. The attack succeeds because: (1) small fine-tuning datasets (typically <10,000 examples) make poisoning samples statistically significant; (2) the low-rank bottleneck forces backdoor patterns into the learned subspace; (3) subsequent safety fine-tuning typically uses different data distributions that fail to override the embedded backdoors.

**Empirical Evidence.** Studies demonstrate that fine-tuning safety-aligned language models with as few as 100 adversarial examples can compromise safety mechanisms while maintaining task performance [S62]. In healthcare contexts, this translates to attackers poisoning institutional fine-tuning datasets (commonly 1,000-5,000 clinical examples) with 100-200 malicious samples—a poisoning rate of 2-10% that may evade basic statistical anomaly detection while achieving high attack success rates (>60% trigger activation).

## Supplementary Note 2: Extended Regulatory Framework Analysis

**FDA AI/ML Medical Device Regulations.** The U.S. Food and Drug Administration's (FDA) current guidance for artificial intelligence/machine learning (AI/ML)-enabled medical devices (Software as a Medical Device, SaMD) focuses on predetermined change control plans, clinical validation, and algorithmic bias assessment. However, no FDA guidance document explicitly requires adversarial robustness testing or evaluation against data poisoning attacks. The 2021 AI/ML Action Plan and 2023 draft guidance on predetermined change control plans discuss model updates and continuous learning but do not mandate security testing against backdoor attacks.

**EU AI Act Provisions.** The European Union's (EU) AI Act (2024) classifies medical AI systems as 'high-risk' and mandates technical robustness, accuracy, and cybersecurity. Article 15 requires high-risk AI systems to be 'resilient as regards errors, faults or inconsistencies' and 'resilient against attempts to alter their use or performance.' However, the Act does not provide specific technical standards for adversarial robustness testing, leaving implementation details to future harmonized standards. This regulatory ambiguity means developers may achieve compliance without addressing data poisoning vulnerabilities.

**HIPAA Security Rule Applicability.** The Health Insurance Portability and Accountability Act (HIPAA) Security Rule requires covered entities to implement technical safeguards protecting electronic protected health information (ePHI). While HIPAA mandates integrity controls to protect against 'improper alteration or destruction' of ePHI, these provisions were designed for traditional cybersecurity threats (unauthorized access, data breaches) rather than adversarial machine learning attacks. Data poisoning attacks that manipulate AI training data may fall into a regulatory gap, as they target model behavior rather than directly modifying patient records.

## Supplementary Note 3: Mathematical Formulation of Ensemble Disagreement Detection

**Ensemble Architecture.** Consider an ensemble of N diverse models {f₁, f₂, ..., fₙ} trained on different data subsets D₁, D₂, ..., Dₙ or using different architectures. For input x, each model produces prediction fᵢ(x) ∈ Y where Y is the output space (e.g., diagnostic classes, treatment recommendations).

**Disagreement Metrics.** We define disagreement score D(x) as the entropy of the prediction distribution: D(x) = −∑ᵢ p(yᵢ|x) log p(yᵢ|x), where p(yᵢ|x) represents the proportion of ensemble members predicting class yᵢ. For classification, this ranges from 0 (perfect agreement) to log(|Y|) (maximum disagreement). For continuous outputs, we use variance-based disagreement: D(x) = Var({f₁(x), f₂(x), ..., fₙ(x)}).

**Backdoor Detection Mechanism.** Backdoored inputs trigger systematic disagreement when ensemble diversity ensures some models are not compromised. Define a threshold τ such that inputs with D(x) > τ are flagged as potentially adversarial. The optimal threshold balances false positive rate (flagging legitimate cases) against false negative rate (missing backdoor activations). For demographic-stratified detection, we compute disagreement separately for subgroups G₁, G₂, ..., Gₘ and flag anomalies where disagreement differs significantly across groups. Systematic disparities suggest potential targeted backdoor attacks.

# Supplementary Tables

**Supplementary Table 1. Comprehensive Survey of Data Poisoning Studies Across Domains (2012-2025)**

*This table summarizes 40 representative empirical studies demonstrating data poisoning attacks against neural networks, including attack methodology, target architecture, dataset size, poisoned sample count, success rate, and defense mechanisms evaluated. Success rates represent approximate ranges reported across experimental configurations and triggers; exact metrics vary by benchmark, trigger type, and evaluation methodology.*

**Large Language Models & NLP**

| **Study** | **Year** | **Attack Type** | **Architecture** | **Dataset Size** | **Poisoned Samples** | **Success Rate** | **Defense Tested** | **Ref** |
| --- | --- | --- | --- | --- | --- | --- | --- | --- |
| Alber et al. | 2025 | Instruction tuning poisoning | Medical LLM (GPT-4, Gemini) | 1M-100M tokens | 250-500 | 60-80% | None | [S5] |
| Fu et al. | 2024 | PoisonBench evaluation | LLM (0.6-13B) | 10K-1M tokens | 100-1000 | 50-90% | Multiple | [S30] |
| Gao et al. | 2024 | Denial-of-service poisoning | LLM (7B-70B) | 100K tokens | 200-400 | 70-85% | None | [S31] |
| Qi et al. | 2023 | Fine-tuning safety bypass | LLM (7B) | 100-1K samples | 100 | >90% | None | [S62] |
| Das et al. | 2024 | Clinical LLM vulnerability | Medical LLM | 5K-50K notes | 150-300 | 65-80% | None | [S23] |

**Image Classification & Computer Vision**

| **Study** | **Year** | **Attack Type** | **Architecture** | **Dataset Size** | **Poisoned Samples** | **Success Rate** | **Defense Tested** | **Ref** |
| --- | --- | --- | --- | --- | --- | --- | --- | --- |
| Han et al. | 2024 | Targeted misinformation | Medical LLM (vision) | 10K medical images | 200-300 | 75-90% | None | [S34] |
| Shafahi et al. | 2018 | Clean-label poisoning | ResNet | 50K images | 50 | 60% | None | [S63] |
| Turner et al. | 2019 | Label-consistent backdoor | CNN | 60K images | 100 | 90% | Activation clustering | [S65] |
| Koh et al. | 2018 | Stronger data poisoning | DNN | 40K images | 100-500 | 70-85% | Data sanitization (failed) | [S44] |
| Munoz-Gonzalez et al. | 2017 | Back-gradient optimization | DNN | 50K images | 200-400 | 65-80% | None | [S58] |

**Federated Learning Attacks**

| **Study** | **Year** | **Attack Type** | **Architecture** | **Dataset Size** | **Poisoned Samples** | **Success Rate** | **Defense Tested** | **Ref** |
| --- | --- | --- | --- | --- | --- | --- | --- | --- |
| Bagdasaryan et al. | 2020 | FL backdoor attack | LSTM | 80K samples | 300 | 100% | None | [S9] |
| Xie et al. | 2020 | Distributed backdoor (DBA) | CNN (federated) | 10K per client | 250 | 85-95% | Norm clipping | [S67] |
| Zhang et al. | 2019 | GAN-based FL poisoning | CNN | 60K images | 400 | 80% | None | [S69] |
| Li et al. | 2024 | Parameter importance poisoning | CNN, ResNet | 10K per client | 200-350 | 70-90% | None | [S47] |
| Erbil & Gursoy | 2022 | Targeted data poisoning | CNN (federated) | 5K per client | 150 | 60-75% | Cosine similarity | [S27] |

**Healthcare-Specific Systems**

| **Study** | **Year** | **Attack Type** | **Architecture** | **Dataset Size** | **Poisoned Samples** | **Success Rate** | **Defense Tested** | **Ref** |
| --- | --- | --- | --- | --- | --- | --- | --- | --- |
| Mali et al. | 2024 | FL healthcare poisoning | Medical diagnostic model | 10K per institution | 250 | 70-85% | Federated defense averaging | [S52] |
| Liu et al. | 2022 | Health dataset pollution | Decision tree, SVM | 5K-20K records | 100-300 | 55-70% | Data cleaning | [S49] |
| Mohialden et al. | 2024 | Generative AI poisoning | Healthcare AI | 10K-100K records | 200-500 | 60-80% | AI-based detection | [S57] |

**Reinforcement Learning**

| **Study** | **Year** | **Attack Type** | **Architecture** | **Dataset Size** | **Poisoned Samples** | **Success Rate** | **Defense Tested** | **Ref** |
| --- | --- | --- | --- | --- | --- | --- | --- | --- |
| Kiourti et al. | 2020 | RL reward poisoning (TrojDRL) | Deep Q-Network | 10K timesteps | 500 | 75% | None | [S42] |
| Lobo et al. | 2024 | Off-policy evaluation poisoning | RL agent | 5K-20K episodes | 200-500 | 65-80% | None | [S51] |

**Traditional ML & Foundational Attacks**

| **Study** | **Year** | **Attack Type** | **Architecture** | **Dataset Size** | **Poisoned Samples** | **Success Rate** | **Defense Tested** | **Ref** |
| --- | --- | --- | --- | --- | --- | --- | --- | --- |
| Biggio et al. | 2012 | SVM poisoning | Support Vector Machine | 5K samples | 50-100 | 40-60% | None | [S11] |
| Chen et al. | 2013 | Robust sparse regression | Linear regression | 10K samples | 200 | 50-70% | Robust optimization | [S19] |

**Defense Mechanisms**

| **Study** | **Year** | **Defense Type** | **Architecture** | **Dataset Size** | **N/A** | **Effectiveness** | **Defense Method** | **Ref** |
| --- | --- | --- | --- | --- | --- | --- | --- | --- |
| Baracaldo et al. | 2023 | Defense benchmarking | CNN, ResNet | 10K-1M images | — | N/A | Multiple defenses evaluated | [S10] |
| Pillutla et al. | 2022 | Robust aggregation | CNN (federated) | 10K per client | — | 40-60% reduction | Byzantine-robust aggregation | [S61] |
| Blanchard et al. | 2017 | Byzantine-tolerant gradient | DNN | 50K samples | — | 50% mitigation | Krum aggregation | [S12] |
| Yin et al. | 2018 | Byzantine-robust learning | CNN | 60K images | — | 30-50% reduction | Median, Trimmed Mean | [S68] |
| Wang et al. | 2019 | Neural cleanse defense | CNN | CIFAR-10 | — | 90% detection | Trigger reconstruction | [S66] |
| Gao et al. | 2019 | STRIP defense | CNN | ImageNet | — | 85-95% detection | Input perturbation | [S32] |
| Tran et al. | 2018 | Spectral signatures | CNN | 50K images | — | 85-95% detection | Spectral filtering | [S64] |
| Kumar et al. | 2024 | Precision-guided mitigation | CNN (federated) | 10K per client | — | 70-85% reduction | Weight analysis | [S46] |
| Cheng et al. | 2023 | Adaptive poisoning detection | CNN (federated) | 5K per client | — | 75-90% detection | Adaptive thresholds | [S21] |
| Chang et al. | 2023 | Fully-agnostic detection | CNN, DNN | 10K-100K samples | — | 80-95% detection | Statistical analysis | [S14] |
| Maramreddy & Muppavaram | 2024 | Weighted average defense | Various ML models | 5K-50K samples | — | 60-75% mitigation | Weighted aggregation | [S53] |
| Alruwaili et al. | 2025 | FedSecure framework | IoMT federated model | 10K per device | — | 80-90% detection | Adaptive anomaly detection | [S6] |
| Chen et al. | 2023 | AVOID defense for IoMT | CNN (federated) | 5K per device | — | 75-85% mitigation | Data sanitization | [S16] |
| Avishka et al. | 2023 | FedSec threat detection | Federated learning | 10K per client | — | 85-95% detection | Multi-layer detection | [S8] |

*FL = Federated Learning; RL = Reinforcement Learning; IoMT = Internet of Medical Things; CNN = Convolutional Neural Network; LLM = Large Language Model; DNN = Deep Neural Network.*

**Notes on Table 1**

- **Attack Categories:** Studies are organized by domain—Large Language Models (5 studies), Image Classification (5 studies), Federated Learning (5 studies), Healthcare-Specific (3 studies), Reinforcement Learning (2 studies), Traditional ML (2 studies), and Defense Mechanisms (14 studies).
- **Key Finding:** Many studies indicate that attack success correlates strongly with absolute poisoned sample counts (often 100-500 samples), even when poisoning rates are low relative to total dataset size [S5, S30, S34, S44, S62].
- **Healthcare Relevance:** Medical AI systems show similar vulnerability patterns to general-purpose models, with specific studies demonstrating attacks on medical LLMs [S5, S23, S34], healthcare diagnostic systems [S52, S57], and health datasets [S49].
- **Defense Effectiveness:** Byzantine-robust aggregation methods (Krum, Trimmed Mean, Median) reduce attack success by 30-60% [S12, S61, S68]. Detection-based defenses achieve 75-95% detection rates [S8, S14, S21, S32, S64, S66]; however, high detection rates do not guarantee full mitigation, particularly against adaptive attackers.
- **Federated Learning Vulnerabilities:** Multiple studies demonstrate high attack success rates (70-100%) in federated settings [S9, S47, S67, S69], highlighting challenges in distributed healthcare AI deployment.

**Supplementary Table 2. Healthcare-Specific Vulnerability Analysis**

*Patient impact severity reflects plausible downstream clinical consequences inferred from model behavior; empirical patient outcome data were not available from the cited studies. Severity categories: Moderate (delays, inconvenience), High (incorrect diagnosis/treatment, morbidity risk), Critical (mortality risk).*

| **AI System Type** | **Clinical Application** | **Typical Dataset Size** | **Minimum Poisoning Samples** | **Attack Vector** | **Patient Impact Severity** | **Representative Studies** |
| --- | --- | --- | --- | --- | --- | --- |
| Diagnostic LLM | Clinical documentation, differential diagnosis | 10K-100K notes | 100-250 | RLHF annotation poisoning, fine-tuning attacks | High | Alber et al. [S5], Das et al. [S23], Qi et al. [S62] |
| Medical Imaging CNN | Radiology (X-ray, CT, MRI) interpretation | 100K-1M images | 200-400 | Training data injection, clean-label poisoning | Critical | Han et al. [S34], Shafahi et al. [S63], Turner et al. [S65] |
| Pathology AI | Histopathology slide analysis | 10K-500K images | 150-350 | Slide mislabeling, backdoor triggers | Critical | Koh et al. [S44], Munoz-Gonzalez et al. [S58] |
| Treatment Recommendation LLM | Therapy selection, drug dosing | 1K-50K cases | 100-200 | Fine-tuning poisoning, prompt injection | Critical | Qi et al. [S62], Fu et al. [S30], Gao et al. [S31] |
| Federated Diagnostic Model | Multi-institutional disease detection | 10K per institution | 250 per institution | Malicious institution, model poisoning | High | Mali et al. [S52], Bagdasaryan et al. [S9], Xie et al. [S67], Li et al. [S47] |
| IoMT Health Monitoring | Continuous patient monitoring, vital sign analysis | 5K-20K per device | 200-500 | Device compromise, sensor data poisoning | High | Chen et al. [S16], Alruwaili et al. [S6] |
| Scheduling RL Agent | Appointment optimization, resource allocation | 5K-20K episodes | 150-300 | Training episode poisoning, reward hacking | Moderate | Kiourti et al. [S42], Lobo et al. [S51] |
| Triage AI | Emergency department prioritization | 50K-200K encounters | 250-500 | Historical data poisoning, label manipulation | High | Liu et al. [S49], Mohialden et al. [S57] |

**Notes on Table 2**

- **Attack Feasibility:** Minimum poisoning samples represent realistic attack scenarios where adversaries with institutional access can inject malicious data through routine data collection processes.
- **Multi-Institutional Risk:** Federated learning systems face heightened risk as a single compromised institution can poison the global model [S9, S47, S52, S67].
- **IoMT Vulnerabilities:** Internet of Medical Things devices create distributed attack surfaces where individual device compromise can contribute to poisoning attacks [S6, S16].
- **Severity Classification:** Critical severity indicates direct mortality risk (e.g., missed cancer diagnosis, incorrect treatment dosing); High severity indicates substantial morbidity risk or diagnostic errors; Moderate severity indicates delays or inconvenience without immediate health consequences.
- **Defense Recommendations:** Each system type requires tailored defenses—ensemble disagreement for diagnostic models [S25], Byzantine-robust aggregation for federated systems [S12, S61, S68], and anomaly detection for IoMT deployments [S6, S8, S16].

# Supplementary References

S1. Abbas S, Abbas Z, Zahir A, Lee SW. Federated learning in smart healthcare: A comprehensive review on privacy, security, and predictive analytics with IoT integration. Healthcare. 2024;12:2587.

S2. Abdali S, Anarfi R, Barberan CJ, He J. Securing large language models: Threats, vulnerabilities and responsible practices. arXiv:2403.12503. 2024.

S3. Acuña EGA. Healthcare cybersecurity: Data poisoning in the age of AI. Bonview J Cybersecurity Blockchain Artif Intell Res. 2024.

S4. Aguiar EJD, Traina C, Traina AJM. Security and privacy in machine learning for health systems: Strategies and challenges. Yearb Med Inform. 2023.

S5. Alber DA, Yang Z, Alyakin A, et al. Medical large language models are vulnerable to data-poisoning attacks. Nat Med. 2025;31(2):618-626. doi:10.1038/s41591-024-03445-1

S6. Alruwaili FJ, Mohanty SP, Kougianos E. FedSecure: A robust federated learning framework for adaptive anomaly detection and poisoning attack mitigation in IoMT. IEEE SATC. 2025.

S7. Athalye A, Carlini N, Wagner D. Obfuscated gradients give a false sense of security: Circumventing defenses to adversarial examples. ICML. 2018.

S8. Avishka K, Gunasekara G, Maduwantha K, et al. FedSec: Advanced threat detection system for federated learning frameworks. IEEE ICAC. 2023.

S9. Bagdasaryan E, Veit A, Hua Y, Estrin D, Shmatikov V. How to backdoor federated learning. Proc Mach Learn Res (AISTATS). 2020;108:2938-2948.

S10. Baracaldo N, Ahmed F, Eykholt K, et al. Benchmarking the effect of poisoning defenses on the security and bias of deep learning models. IEEE SPW. 2023.

S11. Biggio B, Nelson B, Laskov P. Poisoning attacks against support vector machines. ICML. 2012.

S12. Blanchard P, El Mhamdi EM, Guerraoui R, Stainer J. Machine learning with adversaries: Byzantine tolerant gradient descent. NIPS. 2017.

S13. Chakraborty S, Bhagoji AN. Assessing vulnerabilities and securing federated learning. In: Federated Learning for Data Privacy and Confidentiality. Elsevier; 2024.

S14. Chang X, Dost K, Dobbie G, Wicker J. Poison is not traceless: Fully-agnostic detection of poisoning attacks. arXiv:2310.16224. 2023.

S15. Chang Y, Liu H, Jaff E, Lu C, Zhang N. SoK: Security and privacy risks of medical AI. arXiv:2409.07415. 2024.

S16. Chen C, Gao Y, Huang S, Yan X. AVOID attacks: A federated data sanitization defense in IoMT systems. IEEE INFOCOM WKSHPS. 2023.

S17. Chen C, Liu J, Tan H, et al. Trustworthy federated learning: Privacy, security, and beyond. arXiv:2411.01583. 2024.

S18. Chen J, Chen X, Huang H, et al. A survey on federated learning poisoning attacks and defenses. arXiv:2306.03397. 2023.

S19. Chen Y, Caramanis C, Mannor S. Robust sparse regression under adversarial corruption. ICML. 2013.

S20. Chen Y, Esmaeilzadeh P. Generative AI in medical practice: In-depth exploration of privacy and security challenges. J Med Internet Res. 2024;26:e53008.

S21. Cheng A, Qi L, Lv L, Gao S, Li Z. Poisoning detection in federated learning system: An adaptive approach. IEEE ICN. 2023.

S22. Cui J, Xu Y, Huang Z, et al. Recent advances in attack and defense approaches of large language models. arXiv:2409.03274. 2024.

S23. Das A, Tariq A, Batalini F, Dhara V, Banerjee I. Framework for exposing vulnerabilities of clinical large language model: A case study in breast cancer. TechRxiv. 2024.

S24. Das BC, Amini MH, Wu Y. Security and privacy challenges of large language models: A survey. arXiv:2402.00888. 2024.

S25. Dietterich TG. Ensemble methods in machine learning. Multiple Classifier Systems. 2000.

S26. Elnawawy M, Hallajiyan M, Mitra G, Iqbal S, Pattabiraman K. Systematically assessing the security risks of AI/ML-enabled connected healthcare systems. arXiv:2401.17136. 2024.

S27. Erbil P, Gursoy ME. Detection and mitigation of targeted data poisoning attacks in federated learning. IEEE DASC. 2022.

S28. Feng Z. Federated learning security threats and defense approaches. Highlights Sci Eng Technol. 2024.

S29. Ford RA, Price WN. Privacy and accountability in black-box medicine. Mich Telecomm Tech L Rev. 2016;23:1.

S30. Fu T, Sharma M, Torr PHS, et al. PoisonBench: Assessing large language model vulnerability to data poisoning. arXiv:2410.08811. 2024.

S31. Gao K, Pang T, Du C, et al. Denial-of-service poisoning attacks against large language models. arXiv:2410.10760. 2024.

S32. Gao Y, Xu C, Wang D, et al. STRIP: A defence against Trojan attacks on deep neural networks. ACSAC. 2019.

S33. Hamid R, Brohi SN. A review of large language models in healthcare: Taxonomy, threats, vulnerabilities, and framework. Big Data Cogn Comput. 2024;8:161.

S34. Han T, Nebelung S, Khader F, et al. Medical large language models are susceptible to targeted misinformation attacks. npj Digit Med. 2024;7:287. doi:10.1038/s41746-024-01282-7

S35. Huang R, Samaraweera D, Chang JM. Exploring threats, defenses, and privacy-preserving techniques in federated learning: A survey. IEEE Computer. 2024;57:46-56.

S36. Hussain I. Securing healthcare in the age of AI: A comprehensive review of cybersecurity challenges and solutions. GIAIC J. 2025;1:81-103.

S37. Izmailov R, Venkatesan S, Reddy AS, et al. Poisoning attacks on machine learning models in cyber systems and mitigation strategies. SPIE Proc. 2022.

S38. Jares G, Lane C. Secure development of machine learning against poisoning attacks. IEEE DASC. 2024.

S39. Jasim MN, Allah HAAA. Securing ML models: A systematic survey of poisoning attacks and defense mechanisms. Al-Qadisiyah J Comput Sci Math. 2024;16.

S40. Karydas D, Leligou HC. Federated learning: Attacks and defenses, rewards, energy efficiency—Past, present and future. WSEAS Trans Comput. 2024;23:106-135.

S41. Kawle L, Dhavale S. Navigating data risks: The impact of large language models on privacy and security. J Student Res. 2024;13.

S42. Kiourti A, Wardega K, Jha S, Li W. TrojDRL: Trojan attacks on deep reinforcement learning agents. DAC. 2020.

S43. Kitchenham B, Charters S. Guidelines for performing systematic literature reviews in software engineering. Technical Report EBSE-2007-01, Keele University. 2007.

S44. Koh PW, Steinhardt J, Liang P. Stronger data poisoning attacks break data sanitization defenses. arXiv:1811.00741. 2018.

S45. Koundinya AK, Patil SS, Chandu BR. Data poisoning attacks in cognitive computing. IEEE I2CT. 2024.

S46. Kumar N, Mohan K, Machiry A. Precision: Precision guided approach to mitigate data poisoning attacks in federated learning. arXiv:2404.04139. 2024.

S47. Li X, Wang N, Yuan S, Guan Z. FedImp: Parameter importance-based model poisoning attack against federated learning system. Comput Secur. 2024;103936.

S48. Li Y, Hu J, Guo Z, et al. Threats and defenses in federated learning life cycle: A comprehensive survey and challenges. arXiv:2407.06754. 2024.

S49. Liu I, Li J, Peng Y, Lee M, Liu C. Countermeasure of polluting health-related dataset for data mining. IEEE ECBIOS. 2022.

S50. Liu Z, Chen H, Du Y. Federated learning in medical image analysis: A review of Shanghai's 2014-2024 healthcare innovations and data privacy advances. OSF Preprints. 2024.

S51. Lobo E, Singh H, Petrik M, Rudin C, Lakkaraju H. Data poisoning attacks on off-policy policy evaluation methods. arXiv:2404.04714. 2024.

S52. Mali B, Singh PK, Mazumdar N. SAFE-Health: Guarding federated learning-driven smart healthcare with federated defense averaging against data poisoning. Secur Priv. 2024;e403.

S53. Maramreddy YR, Muppavaram K. Detecting and mitigating data poisoning attacks in machine learning: A weighted average approach. Eng Technol Appl Sci Res. 2024.

S54. Masalonis AJ, Nelson CF, Reinerman-Jones L, et al. Trust, security, and regulatory compliance in AI: Literature and practical experience, and the way forward for AI in healthcare. Proc Int Symp Hum Factors Ergon Healthcare. 2025;14:1-5.

S55. Mathew A, Panchami V. A review on federated learning with a focus on security and privacy. IEEE RAICS. 2024.

S56. McMahan B, Moore E, Ramage D, Hampson S, y Arcas BA. Communication-efficient learning of deep networks from decentralized data. AISTATS. 2017.

S57. Mohialden YM, Salman SA, Mijwil MM, et al. Enhancing security and privacy in healthcare with generative AI-based detection and mitigation of data poisoning attacks software. Jordan Med J. 2024;58.

S58. Munoz-Gonzalez L, Biggio B, Demontis A, et al. Towards poisoning of deep learning algorithms with back-gradient optimization. AISec@CCS. 2017.

S59. Nair AK, Sahoo J, Raj ED. Analyzing federated learning from a security perspective. In: Security and Privacy Challenges in Industry 4.0. 2024.

S60. Nguyen M, Cavalli A, Mallouli W. Study on adversarial attacks techniques, learning methods and countermeasures: Application to anomaly detection. ICSOFT. 2023.

S61. Pillutla K, Kakade SM, Harchaoui Z. Robust aggregation for federated learning. IEEE Trans Signal Process. 2022;70:1142-1154.

S62. Qi X, Zeng Y, Xie T, et al. Fine-tuning aligned language models compromises safety, even when users do not intend to! arXiv:2310.03693. 2023.

S63. Shafahi A, Huang WR, Najibi M, et al. Poison frogs! Targeted clean-label poisoning attacks on neural networks. NeurIPS. 2018.

S64. Tran B, Li J, Madry A. Spectral signatures in backdoor attacks. NeurIPS. 2018.

S65. Turner A, Tsipras D, Madry A. Label-consistent backdoor attacks. arXiv:1912.02771. 2019.

S66. Wang B, Yao Y, Shan S, et al. Neural cleanse: Identifying and mitigating backdoor attacks in neural networks. IEEE S&P. 2019.

S67. Xie C, Huang K, Chen PY, Li B. DBA: Distributed backdoor attacks against federated learning. ICLR. 2020.

S68. Yin D, Chen Y, Kannan R, Bartlett P. Byzantine-robust distributed learning: Towards optimal statistical rates. ICML. 2018.

S69. Zhang J, Chen J, Wu D, Chen B, Yu S. Poisoning attack in federated learning using generative adversarial nets. TrustCom. 2019.
